# Community-level socioeconomic inequality in the incidence of ischemic heart disease: a nationwide cohort study

**DOI:** 10.1186/s12872-020-01389-1

**Published:** 2020-02-22

**Authors:** Jun Gyo Gwon, Jimi Choi, Young Jin Han

**Affiliations:** 1 Department of Transplantation and Vascular Surgery, Korea University College of Medicine, Seoul, Korea; 2 Department of Biostatistics, Korea University College of Medicine, Seoul, Korea; 3 Department of Vascular Surgery, Ulsan University College of Medicine and Asan Medical Center, 88, Olympic-ro 43-gil, Songpa-gu, Seoul, 05505 Korea

**Keywords:** Epidemiology, Socioeconomic status, Ischemic heart disease, Health risk behaviors

## Abstract

**Background:**

The purpose of this study was to confirm that inequalities in community-level social economic status (SES) do actually impact the incidence of ischemic heart disease (IHD) using the Korean population-based cohort study of the National Health Insurance Service–National Sample Cohort (NHIS-NSC) database.

**Methods:**

This study used the NHIS-NSC database, a population-based cohort database established by the NHIS in South Korea. Community-level SES was classified into three categories, i.e. low, moderate, and high, according to the rank. The outcome measure of interest was IHD, which was defined according to the International Classification of Disease, 10th Revision (ICD-10) codes.

**Results:**

In the low community-level SES group, the incidence of IHD was 3.56 per 1000 person years (cumulative incidence rate, 1.78%), and in the high community level SES group, it was 3.13 per 1000 person years (cumulative incidence rate, 1.57%). Multivariate analysis showed that the incidence of IHD was higher in the low community-level SES group (*p* = 0.029). The log-rank test showed that the cumulative incidence of IHD was higher in the low community level SES group than the high community-level SES group (adjusted hazard ratio, 1.16; 95% CI, 1.01–1.32).

**Conclusions:**

People living in areas with low community-level SES show an increased incidence of IHD. Therefore, intervention in active, health-risk behavior corrections at the local level will be required to reduce the incidence of IHD.

## Background

Ischemic heart disease (IHD) encompasses the diagnoses of angina pectoris, myocardial infarction, silent myocardial ischemia, and mortality that results from coronary artery disease [1]. Many studies have previously reported the association of IHD with individual-level social economic status (SES). These studies have shown that the lower the individual SES, the higher the incidence of IHD [2–5]. Previous studies have reported that individual lack of awareness of both the risk factors for IHD and the health risk behaviors associated with IHD underlie these findings [5, 6]. Local community influences this lack of awareness for risk factors for IHD and health risk behaviors associated with IHD, which include smoking, consumption of diets with high sodium and high content of trans-fats and low content of polyunsaturated fatty acids, consumption of sugar-sweetened beverages, alcohol abuse, physical inactivity, and psychological stress [7–9]. However, to the best of our knowledge, no study has investigated whether differences in community-level SES affect the incidence of IHD. Therefore, the purpose of this study is to confirm that inequalities in community-level SES have an impact on the incidence of IHD through analysis of the Korean population-based cohort study of the National Health Insurance Service–National Sample Cohort (NHIS-NSC) database.

## Methods

### Study population

**Fig. 1:**
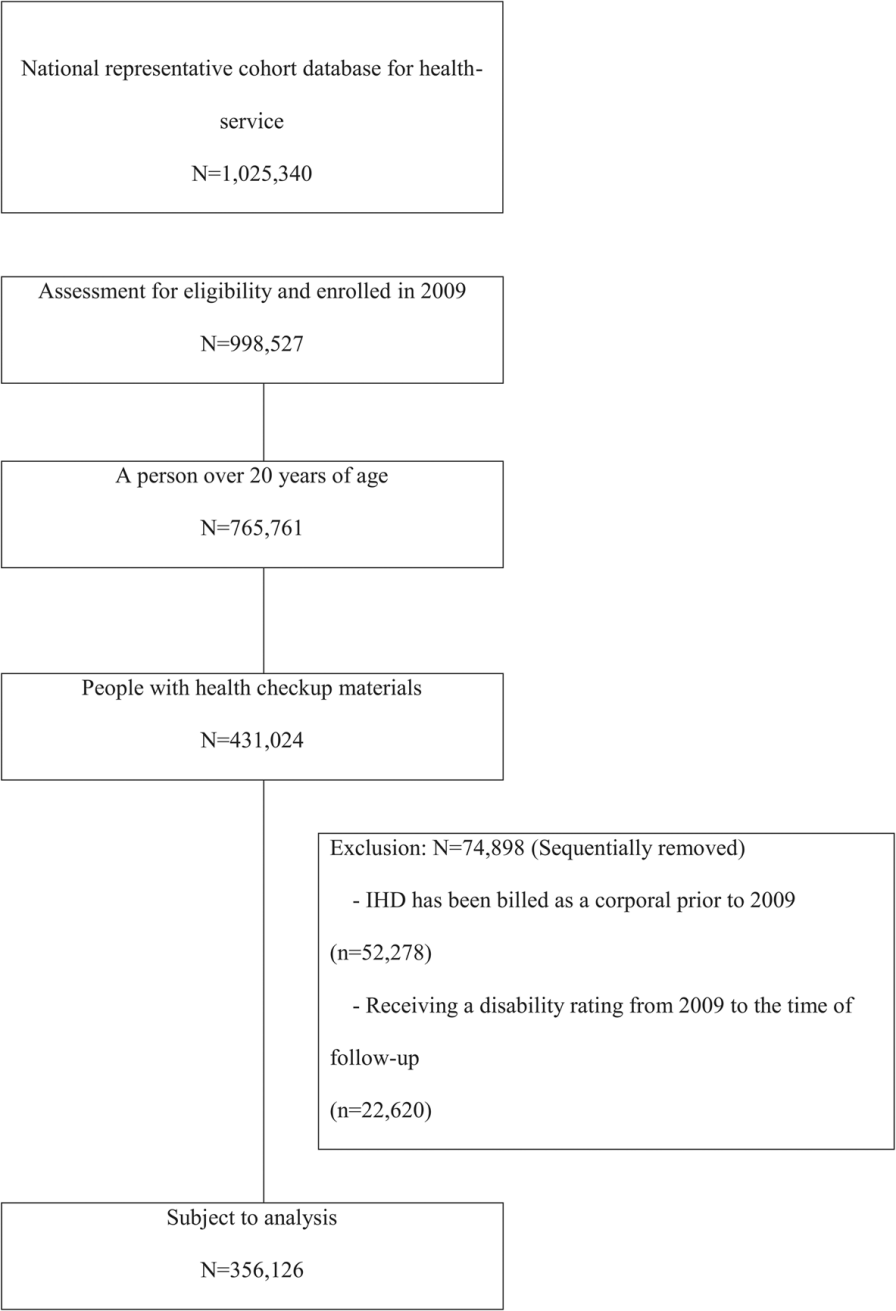
Flowchart of the study

This study used the NHIS–NSC database (NHIS-2018-2-290), a population-based cohort database established by the NHIS in South Korea [10]. This is the national representative cohort database for health service use, in which approximately 1,025,340 patients (2.2% of 46,605,433 Korean residents in 2002) were followed up until 2013 by annually updating samples of newborn infants. From this database, all patients aged ≥20 years in 2009 were identified. Patients without health check-up data or those with a history of ischemic heart disease before their enrollment were excluded (Fig. 1).

### Exposure measures

Community-level SES of study subjects was defined as the local income for the residential area in which they lived in 2009. The gross regional domestic product (GRDP) per capita of 16 regions (seven metropolitan cities, including the Korean capital, and nine provinces) was used to measure the local income of subjects’ residential area and ranked according to the GRDP [11]. Local income was then classified into three categories according to the ranking of GDPR per capita as low (ranks 12–16), moderate (9–11), and high (1–8) (Table 1). Each of the three categories contained a different number of regions as the total population was divided evenly into three categories.

**Table 1:** The gross regional domestic product per capita and Rank in 2009

District in South Korea	Regions	Gross regional domestic product per capita in 2009 (Unit: 10^6^ KRW)	Rank
Metropolitan city	Seoul (Capital)	26.9	5
	Busan	17.4	13
	Daegu	14.5	16
	Incheon	19.9	9
	Gwangju	16.1	15
	Daejeon	16.9	14
	Ulsan	47.9	1
Province	Gyeonggi	20.8	8
	Gangwon	19.6	10
	Chungbuk	23.0	7
	Chungnam	35.2	2
	Jeonbuk	19.4	11
	Jeonnam	28.9	3
	Gyeongbuk	27.7	4
	Gyeongnam	26.1	6
	Jeju	18.9	12

### Outcome measures

The outcome measure of interest was IHD, defined as according to the International Classification of Disease, 10th Revision (ICD-10) codes I20, I21, I22, I23, I24, and I25 [12]. Follow-up of all patients began on January 1, 2009, and ended when any of the following occurred: onset of ischemic heart disease, death from any cause, moving to a different region at baseline, and the end of the study period (December 31, 2013).

### Confounding variables

Confounding variables evaluated included patients’ age, sex, individual economic status, smoking status, body mass index (BMI) and the incidence of comorbidities including diabetes mellitus (DM), hypertension (HTN), dyslipidemia, peripheral arterial disease (PAD), and stroke at baseline. The people included in the NHIS are ranked into 21 categories on the NHIS-NSC database according to the insurance premiums that they pay. The NHIS calculates individual insurance premiums through consideration of income, assets, standard of living, and other economic factors. In our statistical modelling, individual economic status was evaluated as the average premium value for the insurance premiums in each ranks of NHIS. The history of disease was defined as follows: DM (ICD-10 E11, E12, E13, E14), HTN (I10, I11, I12, I13, I15), dyslipidemia (ICD-10 E78), PAD (ICD-10 I70.0, I70.2, I73.9, I70.8, I70.9, I74.2, I74.3, I74.4, I74.5), and stroke (ICD-10 I60, I61, I63).

### Statistical analyses

Data are presented as means (standard deviation, SD) for continuous variables and as numbers (n) and percentages (%) for categorical variables. Demographic and clinical characteristics among the regional income group were compared using the chi-square test or ANOVA, as appropriate. The incidence rate per 1000 person-years and cumulative incidence for IHD were calculated in each group. To evaluate the association between the risk of IHD and regional income level, Cox regression models with mixed effect (“Frailty model”) were used. This model incorporates region-specific random effects to account for within-region homogeneity in outcomes [13]. Hazard ratios and 95% confidence intervals were presented, and the high-income group was considered the reference group. Adjusted hazard ratios were obtained from the model including regions as random effect and age, sex, smoking, BMI, individual economic status, history of DM, HTN, dyslipidemia, PAD, and stroke as covariates. All statistical analyses were performed using SAS version 9.4 software (SAS Institute Inc., Cary, NC, USA), and a two-sided *P*-value< 0.05 was considered statistically significant.

## Results

### Baseline and clinical characteristics of the study population

From January 2002 to December 2009, we identified a total of 356,126 patients, excluding those who had previously been diagnosed with IHD and those who were diagnosed according to a disorder code. Characteristics of the study population are shown in Table 2. Among the risk factors of IHD, the smoking ratio and BMI had higher prevalence rates (*p* = 0.006, *p* = 0.001), whereas DM, HTN, and dyslipidemia had lower prevalence rates (p = 0.006, *p* < 0.001, p < 0.001) in the low community-level SES group. PAD had a higher prevalence rate with lower community-level SES (*p* = 0.044), whereas stroke was not associated with lower community-level SES (*p* = 0.745).

**Table 2:** Baseline characteristics of the study populations

Variables	Total (*N* = 356,126)	Community level SES			*p* value*	*p* for trend
		Low (*N* = 109,632)	Medium (*N* = 117,936)	High (*N* = 128,558)		
Age group, n (%)					<.001	0.758
20~29	42,875 (12.0)	12,801 (11.7)	14,058 (11.9)	16,016 (12.5)		
30~39	77,245 (21.7)	22,052 (20.1)	27,256 (23.1)	27,937 (21.7)		
40~49	98,444 (27.6)	31,356 (28.6)	34,306 (29.1)	32,782 (25.5)		
50~59	73,530 (20.7)	23,945 (21.8)	22,359 (19.0)	27,226 (21.2)		
60~69	40,605 (11.4)	12,541 (11.4)	12,473 (10.6)	15,591 (12.1)		
70~79	19,061 (5.4)	5635 (5.1)	6190 (5.3)	7236 (5.6)		
80~	4366 (1.2)	1302 (1.2)	1294 (1.1)	1770 (1.4)		
Sex, n (%)					<.001	0.135
Male	180,598 (50.7)	54,738 (49.9)	61,160 (51.9)	64,700 (50.3)		
Female	175,528 (49.3)	54,894 (50.1)	56,776 (48.1)	63,858 (49.7)		
Smoking Ever, n (%)	120,972 (34.0)	36,648 (33.4)	41,879 (35.5)	42,445 (33.0)	<.001	0.006
Body mass index (kg/m^2^), mean (SD)	23.4 (3.2)	23.4 (3.2)	23.4 (3.3)	23.3 (3.2)	<.001	0.001
Co-morbidities						
Diabetes mellitus, n (%)	28,745 (8.1)	8764 (8.0)	9321 (7.9)	10,660 (8.3)	0.001	0.006
Hypertension, n (%)	61,831 (17.4)	18,737 (17.1)	20,092 (17.0)	23,002 (17.9)	<.001	<.001
Dyslipidemia, n (%)	25,689 (7.2)	7745 (7.1)	8161 (6.9)	9783 (7.6)	<.001	<.001
Peripheral arterial disease, n (%)	5560 (1.6)	1829 (1.7)	1728 (1.5)	2003 (1.6)	0.001	0.044
Stroke, n (%)	6728 (1.9)	2098 (1.9)	2196 (1.9)	2434 (1.9)	0.658	0.745
Individual economic status (Medical premium: won), mean (SD)	77,526.1 (56,833.9)	72,459.8 (54,259.6)	78,492.6 (56,319.4)	80,960.0 (59,100.8)	<.001	<.001

* *p*-value by the chi-square test or ANOVA

### Incidence of IHD according to the community-level SES

**Fig. 2:**
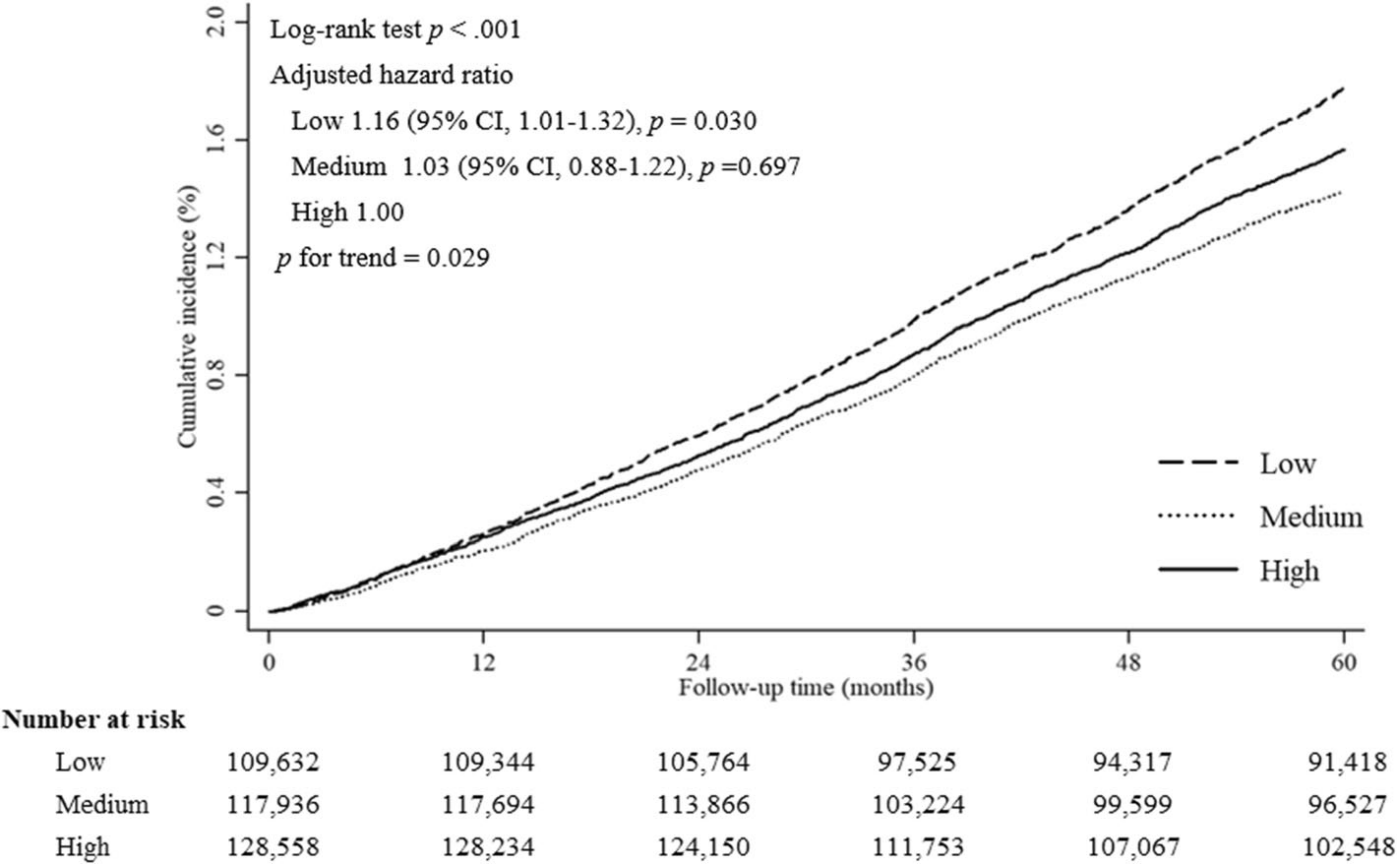
Kaplan-Meier estimates showing the incidence of IHD in the three groups divided by community-level SES

**Fig. 3:**
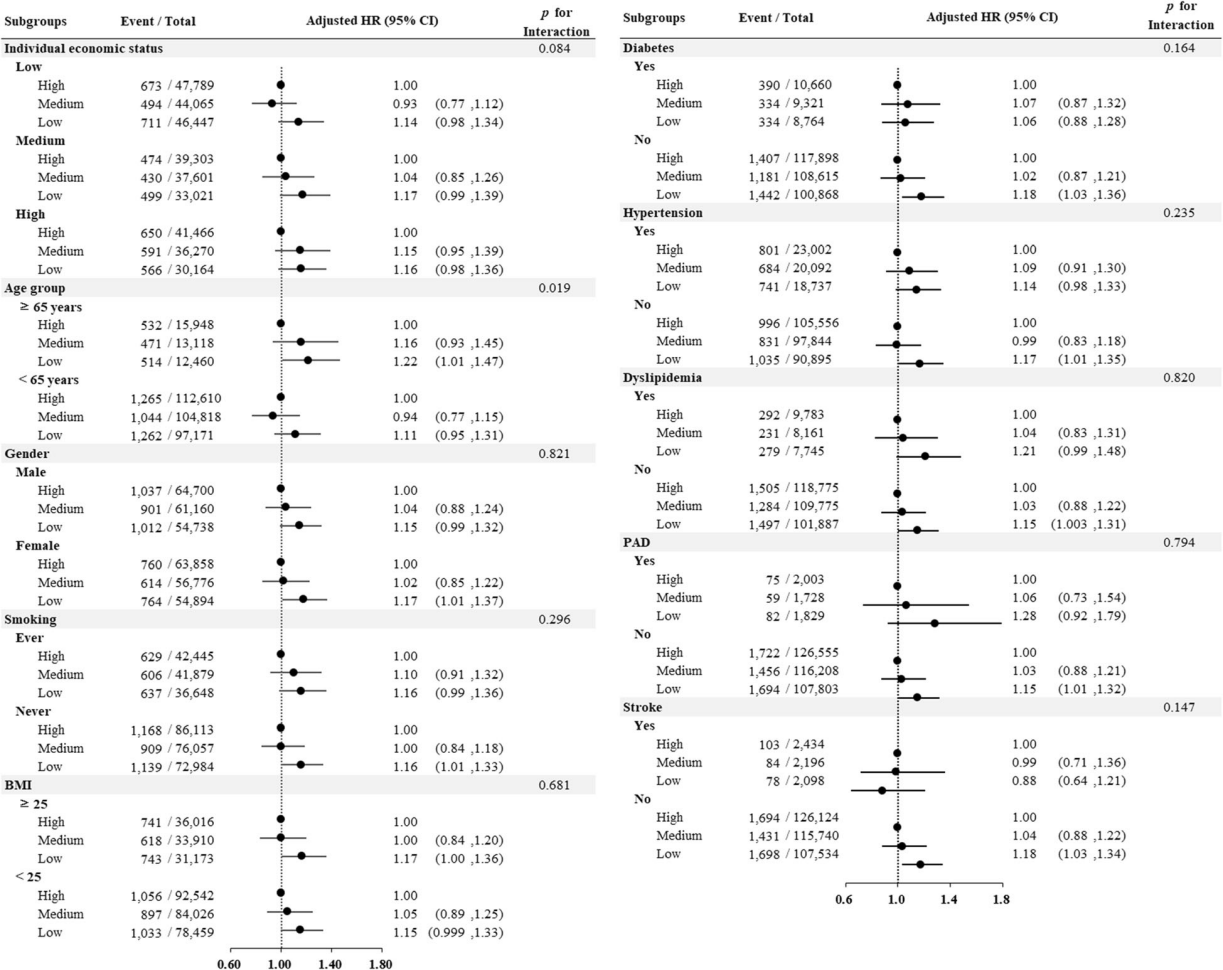
Risk of IHD according to the community-level SES status in the subgroups stratified by other covariates

In the low community-level SES group, the incidence of IHD was 3.56 per 1000 person years (cumulative incidence rate, 1.78%), and in the high community-level SES group, it was 3.13 per 1000 person years (cumulative incidence rate, 1.57%). Multivariate analysis showed that the incidence of IHD was higher in the low community-level SES group (*p* = 0.029, Table 3). Figure 2 shows the cumulative incidence of IHD according to the community-level SES cohort during the follow-up period using the Kaplan-Meier method. The log-rank test showed that the cumulative incidence of IHD was higher in the low community level SES group than in the high community level SES group (adjusted hazard ratio, 1.16; 95% CI, 1.01–1.32). Figure 3 shows the risk of IHD associated with low community-level SES among the various subgroups according to demographic data and comorbidities. The significance of modification effects by each covariate regarding the risk of IHD associated with low community-level SES was also tested, and the results are shown as P for interactions. In the subgroup analysis conducted in the three groups according to individual economic status, individual economic status was shown to not affect the incidence of IHD according to community-level SES (*p* = 0.084). Among the variables included in the subgroup analysis, only patients’ age significantly modified the influence of low community-level SES on the risk of IHD (*p* = 0.019).

**Table 3:** Incidence rates of IHD according to the community level SES status

Community level SES	Event	Total person years	Incidence rate per 1000 person years	Cumulative Incidence^a^ (%)	Crude HR (95% CI)	*p*	Adjusted HR^b^ (95% CI)	*p*
Low	1776	499,273.5	3.56	1.78	1.10 (0.91–1.33)	0.339	1.16 (1.01–1.32)	0.030
Medium	1513	531,663.9	2.84	1.43	0.97 (0.76–1.23)	0.768	1.03 (0.88–1.22)	0.697
High	1797	574,652.6	3.13	1.57	1.00		1.00	
*p* for trend					0.311		0.029	

^a^By Kaplan-Meier’s estimates

^b^Adjusting by age, sex, smoking, body mass index, individual economic status, history of diabetes mellitus, hypertension, dyslipidemia, peripheral arterial disease, and stroke

## Discussion

### Community level SES and IHD

The results of this study confirmed that even though the individual level of SES was adjusted, the incidence of IHD differed according to differences in the community level of SES. The lower the community-level SES, the higher was the incidence of IHD. We hypothesized that different health risk behaviors in different communities may have influenced the incidence of IHD, underlying these findings. Health risk behaviors, such as consumption of diets with high sodium and high levels of trans-fats, alcohol abuse, physical inactivity, and psychological stress all serve as risk factors for IHD and directly affect its incidence [7–9]. These health risk behaviors are influenced by neighbors, and the neighborhood environment is associated with community-level SES positions [14–19]. Mayen et al. reported that individuals consuming a healthier diet are also relatively less frequent in the low SES community [20]. Community-level people with high SES tend to share more information regarding health-related behaviors among neighbors or make an increased effort to reduce risk factors associated with IHD [21, 22]. Based on the results of this study, it is expected that the incidence of IHD will be lowered through community-level interventions such as promoting the use of educational materials on IHD or expanding smoking cessation areas. In fact, some studies have proposed that risk factors for non-communicable diseases, such as IHD, are more likely to be identified at lower SES levels and should, therefore, be controlled at the local level in order to reduce these risk factors [23, 24].

### Advantages of this study

Previous SES-related studies have used income as a reference for SES [25–27]. However, as income does not reflect the level of real estate property, it is unlikely that it is an index that can accurately reflect personal assets. In this study, the government’s medical insurance premium, which is proportional to tax, was used as an indicator of SES. Tax reflects assets more objectively than does income because it also includes the value of owning real estate or an automobile. As this study is a nationwide, register-based cohort study, a higher number of patients were included, along with the use of sample cohort data encompassing the entire nation, ensuring good quality data. Previous studies have mostly been conducted in developed countries with improved well-being [4, 25]; therefore, participants included in those studies would have a relatively lower impact of SES on the medical outcome than most individuals worldwide. For example, in some European countries, where healthcare is provided free of charge, the difference in income will have less impact on the accessibility of health care services. In this respect, this study can more accurately report on the effects of SES inequality on medical outcomes than other studies pertaining to higher income countries. This is because the welfare benefits are relatively less developed and data are obtained from developing countries where SES is unfairly developed due to rapid urbanization [28]. Another advantage of this study is that there are no confounds due to medical differences between the races, as it was conducted in a single nation-state, unlike other nationwide cohort studies.

### Limitations of this study

First, one of the indicators that reflects SES is the education level; however, there is no information regarding education level in this study. Despite this, stratifying patients’ SES according to their educational background was not considered as meaningful, because more than 98% of patients included in this study had high school or higher education and more than 60% received a college education [29]. Second, the diagnosis of the disease may not be accurate. Data used in this study were based on physician’s inputs of the disease, which was classified by ICD-10. Therefore, there is a lack of objective data as to why the physicians in-charge diagnosed the disease. A third limitation was that the results of this study were limited to aged subjects. In subgroup analysis, there was no difference in the incidence of IHD according to community level SES for those aged < 65 years. Therefore, it is difficult to draw conclusions on the different incidence of IHD according to differences in community-level SES in people aged < 65 years. However, because the incidence of IHD is relatively low in young age [30], people aged < 65 years group may not have adequately reflected the incidence of IHD according to the difference of community level SES. In this study, there were also differences in the incidence of IHD between old age group and young age group. The incidence of IHD in people aged ≥65 years was 4.56% (95% CI, 4.34–4.80), and the incidence of IHD in people aged < 65 years was 1.25% (95% CI, 1.21–1.29).

## Conclusions

People living in areas with low community-level SES tend to have an increased incidence of IHD. Therefore, interventions through active, health-risk behavior corrections at the local level should be implemented in order to reduce the incidence of IHD.

## Data Availability

This data is available from the National Health Insurance Service–National Sample Cohort (NHIS-NSC) in Korea. However, NHIS (https://nhiss.nhis.or.kr) approval is required to use this data.

## Abbreviations

BMI: Body mass index
DM: Diabetes mellitus
GRDP: The gross regional domestic product
HTN: Hypertension
ICD-10: International classification of disease, 10th revision
IHD: Ischemic heart disease
NHIS-NSC: National Health Insurance Service–National Sample Cohort
PAD: Peripheral arterial disease
SES: Socioeconomic status
